# Perspectives of Dutch women on premenstrual disorder. A qualitative study exploring women’s experiences

**DOI:** 10.1080/13814788.2023.2166033

**Published:** 2023-01-30

**Authors:** Marijke S. Labots-Vogelesang, Rachel Kooiman-Andringa, Theodora A. M. Teunissen, Antoine L. M. Lagro-Janssen

**Affiliations:** Gender and Women’s Health Unit, Department of Primary and Community Care, Radboud University Medical Centre, Nijmegen, the Netherlands

**Keywords:** Premenstrual disorder, women’s health, acknowledgment, coping, behaviour

## Abstract

**Background:**

Women presenting with Premenstrual Disorder (PMD) to general practitioners (GPs) experience problems with their biopsychosocial functioning. PMD is a disorder consisting of physical and/or mood-based symptoms cyclically occurring with a significant impairment of daily life. Little is known about the symptoms and coping strategies of women with PMD and their experiences with their GPs.

**Objectives:**

This present study aimed to improve understanding of the perspectives of women with PMD, their coping strategies and their expectations of the GP.

**Design:**

Qualitative study with semi-structured interviews.

**Setting:**

In 2017, Dutch women with symptoms of premenstrual disorder were recruited through local newspapers in the town of Nijmegen and the North-Holland region and *via* social media. After checking the inclusion and exclusion criteria, we interviewed 20 women (between 27 and 49 years of age). The interviews took place at a location of the interviewees’ preference.

**Results:**

PMD symptoms can strongly influence the quality of women’s lives. Three themes emerged from our analysis: feelings of having two separate female identities, PMD as a life-controlling condition and different coping strategies. Most women used an active coping strategy. Women with PMD need recognition from their GPs and knowledge of proper treatment.

**Conclusion:**

PMD symptoms can have a high impact on daily life. Women with PMD expressed their need for acknowledgement and a personalised approach from their GP. Greater awareness and knowledge among GPs may be helpful in this.

KEY MESSAGES

- Women with PMD perceived themselves as having two identities: a ‘normal’ real self and an ‘abnormal’ PMD identity that is not their actual self.
- GPs should properly recognise and diagnose symptoms of PMD.
- Women expect proper treatment fitted to their coping strategy.

## Introduction

Premenstrual Disorder (PMD) covers a broad spectrum of physiological and psychological symptoms that may disrupt women’s daily life [1–5]. PMD is an umbrella term covering the continuum of premenstrual distress and the overlap between the diagnostic categories Premenstrual Syndrome (PMS) and the more severe Premenstrual Dysphoric Disorder (PMDD) [2,4,6]. Given this continuum of premenstrual distress, the International Society for Premenstrual Disorders (ISPMD) has advocated the use of the umbrella term of PMD in scientific research for a unified approach to diagnosis and treatment (Box 1)[4,6,7]. Physical, mental and behavioural symptoms characterise PMD during the luteal phase of almost every menstrual cycle [1–5]. The symptoms must be recorded in at least two subsequent menstrual cycles to distinguish between cyclically occurring premenstrual symptoms and non-cyclical psychological or physical disorders. Many women of reproductive age experience mild premenstrual symptoms and a certain degree of discomfort is to be considered mainly physiological. The difference with ‘normal menstrual changes’ is that, in the case of PMD, symptoms must be severe enough to affect daily functioning. Women experiencing a high symptom burden due to PMD may seek help from their general practitioner (GP). If women with PMD experience severe problems and marked limitations in their daily psychosocial functioning, the impairment and lowered quality of life is comparable to a major depressive disorder [4,5,8]. This is supported by increased suicidality to severe PMD symptoms [4]. Although the prevalence of severe PMD among European women is estimated to be four to eight per cent, this condition has received little acknowledgement [9,10]. Treatment strategies suggested for PMD, such as cognitive behavioural therapy, selective serotonin reuptake inhibitors and contraceptives to suppress women’s natural reproductive system, are supported by moderate evidence [11,12].

Box 1. Symptom characteristics of PMD [7].

**Table:** 

	Symptoms occur in ovulatory cycles. The symptoms are not specified—they may be somatic and/or physical. The number of symptoms is not specified. Symptoms are absent (some days) after menstruation and before ovulation. They must recur in the luteal phase. They must be prospectively rated (two cycles minimum). Symptoms should cause significant impairment.^a^

^a^Work, school, social activities, interpersonal relationships and distress (Royal College of Obstetricians and Gynaecologists. Premenstrual syndrome and Management).

Though women may experience severe symptoms and treatment options are only moderately successful, the perspectives of women with PMD in the general population have scarcely been studied. It is unknown to what degree these women are affected by PMD, what the consequences are for their daily lives, how they cope with the distress and what they might expect from their GP [13,14]. To our knowledge, there is no PMD consensus guideline for primary care worldwide.

This is a shortcoming because problems and symptoms are first presented to the GP. Concerning menstrual issues, therefore, GPs play an important role in interpreting and labelling the problem patients present. The GP is also the gatekeeper to other primary and secondary healthcare professionals. Interviews with GPs in the Netherlands have revealed that, in general, they consider physiological hormonal changes and personal sensitivity as critical aetiological factors, preferring an approach of showing women how to deal with symptoms [15]. For GPs, it is vital to know what women experience as invalidating and troublesome for them to optimise care. In this qualitative study, therefore, we investigate what symptoms women with PMD in the general population experience as disabling, what their cognitions about PMD are, what coping strategies they use and what expectations they have of their GP.

## Methods

### Study design

Using a qualitative study design based on grounded theory, we performed in-depth, semi-structured interviews with women with PMD symptoms to examine personal experiences, cognitions, coping and expectations of their GP.

### Study population

Women experiencing premenstrual symptoms were invited to participate in the study through local newspapers in the Nijmegen area and the North-Holland region and (closed) PMS/PMDD Facebook pages and a PMS platform. In the announcement, we described what criteria we used for premenstrual symptoms and invited Dutch and English-speaking women to register for an intake by telephone. In the intake, two experienced researchers from the Department of Primary and Community Care and Gender & Women’s Health at Radboud University Medical Centre (ML, GP researcher; RK, medical Master’s student) verified whether the respondents’ premenstrual symptoms met the criteria in the umbrella definition of PMD according to ISPMD (Table 1). The inclusion criteria were: symptoms that caused significant impairment; symptoms that started after ovulation and ended after menstruation; and symptoms that were recurrent in almost every cycle. Exclusion criteria were: uncertainty whether the present symptoms occurred only in the luteal phase; symptoms that were due to the exacerbation of an underlying disorder, such as migraine, IBS, etc.

**Table 1.** Characteristics of participants.

Participants		*n* = 20
Age (years)	25–35	7
	36–45	8
	46–55	5
	Range: 27–49	
	Mean: 38.6 (SD 7.2)	
Ethnicity	Dutch	19
	Moroccan	1
Marital status	Married/partnership	15
	Single	4
	Widowed	1
Household members	With partner and child(ren)	12
	With partner	3
	Single	3
	Other	2
Education	Secondary school	3
	Secondary vocational education and training	8
	Higher education	9
Occupation	Paid employment	16
	Unemployed	3
	Student	1
Working hours	Full-time	10
	Part-time	7
	Unemployed	3
Place of residence	Large city	8
	Small town	4
	Village or countryside	8
Region	Gelderland	6
	North-Holland	9
	Other regions	5
Starting age PMD (yrs.), approximately	Range 14–43 Mean 27.9 (SD 10.3)	
Self-reported health status	Excellent	3
	Good	10
	Fair	2
	No answer	5
Interview method	Face-to-face	8
	By telephone	12
Respondents recruited *via*	Local newspapers	13
	Social media	12

Thirty-three women responded, three of whom did not meet the inclusion criteria. To capture as many variations as possible, we included twenty-five of these thirty women for an interview by purposive sampling based on maximum variability in age, education level, employment and ethnicity. Five respondents did not respond to the invitation or did not have time to spare. The interviews took place at a location of the interviewees’ preference. After conducting twenty interviews by telephone (*n* = 12) or face-to-face (*n* = 8), we ceased recruiting new participants as we had achieved saturation, which is specified in further detail below [16].

### Data collection

Between March and May 2017, in-depth, semi-structured interviews were performed using an interview guide based on literature and advice of an expert committee (consisting of ML, TT (a GP and a researcher experienced in qualitative studies) and ALJ (a GP and a researcher experienced in qualitative studies)). Prior to the interview, participants gave their written informed consent for the interview and its recording. After completing a general questionnaire about background characteristics, the participants were interviewed about their symptoms, cognitions, coping and experiences with their GP guided by an interview guide (Box 2). This guide was developed based on literature and the advice of an expert committee (ML, TT, ALJ). We plotted the guide in two interviews, which were not included in the study.

### Data analysis

Interviews were anonymised and transcribed verbatim. Only the researchers had access to the original interviews. Two researchers (ML, RK) independently analysed the transcripts to limit the researchers’ interpretation and increase reliability. They used Atlas.ti, version 7.1.5 and used thematic analysis to identify important patterns in the data (themes) [17]. The researchers, therefore, read the transcripts, following open, axial and selective coding to conceptualise the data. To reach consensus, the results were compared and discussed after every five interviews. After 20 interviews, inclusion was discontinued as no more new codes appeared and saturation had been reached. The resulting codes were analysed, clustered and categorised into overarching themes [18]. An example of categorisation is set out in the Supplementary Materials. The researchers discussed all findings with the supervising committee until consensus was reached. We supported our interpretation of the findings with quotations, specifying whether results were raised by few (1–4), some (5–9), many (10–15) or most (16–19) participants. We applied the COREQ criteria for reporting qualitative research [19].

## Results

### General findings

We interviewed 20 women with PMD between 27 and 49 years old, with a median age of 39. Three-quarters of them lived with a partner. Participants included women with and without jobs and from urban, country and regional settings. Women with different education levels took part in the interviews, both face-to-face and by telephone (Table 1).

We first analysed what PMD symptoms women experienced and which ones they perceived as being the most disabling. We categorised the relevant emerging views into two themes: 1. PMD feelings of having separate female identities 2. PMD as a life-controlling condition. Secondly, in the analysis of their opinions on the cause of PMD and how to deal with it, a third theme emerged: differences in coping strategies.

### Theme 1

#### Separate female identities

Although some women said they thought that PMD was part of being a normal, authentic woman, many women described themselves as feeling insecure about their appearance and in their social behaviour as if they were no longer the women they used to be.

*I have … headaches, painful breasts usually, abdominal pain, lower back pain, but worst of all are my mood swings. I always say, and I’ve done so for years, that for three weeks I’m a witch and for one week I’m a nice person. (P5, 44yrs)*

Almost all women mentioned that PMD affected their sense of being a ‘normal’ woman and perceived their PMD feelings as abnormal, not their actual selves. They expressed a loss of who they were, particularly their sense of being good mothers.

*But I don’t know. And then I will feel dreadful and a bad mother … I’m not myself at all. No, I’m a totally different person. After that week I’m myself again, but the thing is, well, it’ll take me another week to the repair all the damage I’ve caused. And sometimes that damage is considerable, with regard to the children, for instance. I’m not a fun mum at those times, you know. (P8, 34 yrs)*

Some women felt guilty because, in the PMD weeks, they could not fulfil the role they preferred to play as mothers and partners.

*I’m just not in a good mood and not sociable and sometimes I’m not a nice mum for my kids. I’ll be irritable and on a short fuse. Yes, sometimes I feel very guilty towards my children and also towards my husband. […] I just want to be a normal family member and just a fun mum for my children. (P20, 32yrs)*

They perceived themselves as having two separate identities. Some women found it difficult to distinguish which of the two identities their behaviour matched.

*But I do notice that, during that period, my partner now calls me an ‘alien’, saying things like, ‘Alien, what did you do with my wife? Give her back to me’. (P18, 37yrs)*

When their symptoms diminished after the onset of menstruation, these women felt they were becoming their usual selves again.

*It takes about three days and then I feel more and more mentally calm. The fourth day, I always feel completely myself again. (P16, 44yrs)*

Women experienced tensions in their relationships as their partners had to deal with someone who was discontinuous in her behaviour and competencies.

Women also appeared to be condemning themselves for lacking patience, attention or kindness. They felt they were failing to fulfil the feminine social roles that were expected of them.

*The family, my partner. At those times they tend to tread very carefully, because they know ‘oh dear’… (P 11, 46 yrs)*

One woman described herself as acting aggressively, like a ‘bitch.’

*I’ve been Googling why I’m such a bitch, while I honestly always want to be a nice person. (P18, 37 yrs)*

### Theme 2

#### A life-controlling condition

Many women considered their PMD symptoms to be life-controlling. The choices they made, their contacts with the people they met and the opportunities they faced were influenced by PMD. During the weeks when they had no symptoms, these women were preparing for or worrying about the next PMD phase.

*I’m actually always thinking about it. And when I feel good, I’m already preoccupied with it, like: ‘Oh dear, I hope I won’t feel bad again’. (P20, 32yrs)*

Some women mentioned a decline in their self-esteem and their self-confidence when their PMD phase started.

*That’s the worst thing […] I'm an experienced person and my work went well, but suddenly I started to ask myself questions […], to doubt things. (P3, 41yrs)*

These alternating periods of feeling good and anxious about misunderstanding and misjudgement impeded their social activities. In addition, many women indicated that their PMD influenced their work activities, affecting their concentration, overview and efficiency during their PMD phase. Half the women decided to work fewer hours or to discontinue work because of PMD. Some women said they often called in sick during PMD.

*I gave up my job over a year ago…, called in sick […] because it was all going pear-shaped […]; I’ve always worked and never been ill. (P11, 46yrs)*

PMD appeared to have such an impact that, along with physical, depressive and negative feelings, even suicidal thoughts were not uncommon among the interviewees. Some women mentioned recurrent thoughts about ending their life or not valuing life because of the affected lives they led. A few women had attempted to commit suicide at least once.

*And so at some point I […] would also feel the urge to end it all. (P4, 49yrs)*

### Theme 3

#### Differences in coping strategies

Approaches for coping with the symptoms of PMD could roughly be grouped into two categories: coping by active approach and coping by avoidance.

#### Coping by active approach

Most women used an active, somatic approach. These women considered PMD primarily an illness and preferred to receive specialist PMD care from a specialised doctor or clinic. Many women had disappointing experiences with the GPs, gynaecologists or psychiatrists they had consulted, feeling that they were not being taken seriously or that the GP showed little symptom recognition and even less knowledge of proper treatment.

*If only there were a specialised department for women with such problems, so you’re really being taken seriously and so you won’t be told: ‘oh well, that’s just part of it [being a woman]’. (P6, 27yrs)*

Women reported they felt they ‘needed to be their own doctor.’ Many women used different ways to improve their situation: they sought distraction, searched for new information online, saw several doctors or therapists and tried numerous medicines or therapies.

*It’s one of the characteristics of PMD: when you’re having these symptoms, you’re just looking for solutions, solutions, […] still searching online at two o‘clock at night: ‘maybe I might do this or perhaps this will work?’ (P8, 34yrs)*

Many women reported that they had actively searched for women who had similar experiences and found it helpful to share their experiences or get recognition from others, which made them feel less alone. Others, however, mentioned that the despondency or hopelessness of their peers had a negative influence on their well-being.

*There’s two sides to it. […] You’re not the only one. […] I get a lot of knowledge from them. On the other hand, though, there’s a lot of women with negative stories, and I'd rather not be confronted with those when I'm in a good mood. (P13, 36yrs)*

These women expressed needing diagnostic tools to determine whether their hormones were causing the symptoms. Above all, they expected their GP to properly recognise and diagnose their symptoms and choose an approach that best suited their cognition and well-being.

#### Coping by avoidance

A smaller part of the women coped by just trying to accept the fact they had PMD. These women considered their PMD symptoms normal and appropriate to physiological hormonal changes. They were putting up with it and added self-mockery or tried to laugh their symptoms off, applying behaviour that reduced PMD distress by distancing themselves from the problem.

*But just to think of these problems ‘Okay, just let them be’ instead of ‘I don’t want this. Here we go again.’[…]. And some level of acceptance helps. So to let it be rather than to fight it. (P12, 41yrs)*

These women were more likely to be surprised by the new PMD phase; some said they had repeatedly underestimated their problems when PMD was over and were overwhelmed with each new period of symptoms.

*You can no longer turn to a doctor because […] they had no answers either. […] Yes, around my period I slightly adjust my lifestyle and I isolate myself somewhat. (P4, 49yrs)*

These women mainly adopted a wait-and-see attitude until it was over, partly due to dissatisfaction with the lack of support from their GP.

*If I don´t mention my problems, I shouldn’t expect that she (my GP) will do anything for me either. (P1, 31yrs)*

All women in general, considered it important that their symptoms were recognised by their GP. They found it helpful and reassuring to label themselves as having PMD and to know that how they felt or behaved was not due to them but due to PMD.

## Discussion

### Main findings

The main findings of this qualitative study are, first of all, that PMD makes women feel that they are not who they really are while they are experiencing symptoms. PMD brings out another person who is different from how they see themselves as women, good mothers and partners. They describe this feeling as having two separate identities. Second, they experience PMD as controlling their lives by disrupting their everyday and social activities. Most women use active coping strategies and some avoidant coping strategies according to their cognitions about PMD. Last, many women have disappointing experiences with the physicians they consult. In the GP’s approach, they fail to meet with adequate symptom recognition, knowledge of options for treatment and willingness to look for an approach that best fits their own views.

### Strengths and limitations

The strengths of our study are the significant variability in age, parity and employment of the study population and the achievement of saturation in our data collection. A major pitfall of a qualitative approach is the interference of subjectivity, which we precluded by independently encoding and analysing all interviews with two experienced researchers and structurally and objectively consulting the supervisors’ committee.

A limitation of our study is the low variety of women’s ethnic backgrounds. It is difficult to reach women from other cultures as they are less likely to respond to announcements, perhaps due to limited literacy skills or to cultural restrictions in discussing premenstrual problems [20]. The way we selected the respondents might have led to selection bias, resulting in an overrepresentation of women with severe PMD symptoms.

### Comparison with existing literature

All women included in this study experienced PMD as a great burden in their daily social life. The psychological symptoms of PMD in particular affected their identity, ranging from persistent distress to suicidal thoughts. This is consistent with findings of studies among patients with chronic or recurrent illnesses who face identity changes because of their illness [4,5,21,22]. Women with PMD in our study are more worried about their role as mothers and partners than about their well-being. These identity changes, therefore, are particularly contingent upon their socially constructed female identity. Relationships with other people are an important aspect of this socially constructed female identity, and for women, this involves greater sensitivity to and connection with other people [23]. What standards and gender stereotypes women will be taught and shown during their socialisation process depends on the times in which they grow up and the subcultures in which they move. In a gender-stereotyped representation, women are supposed to be caring, empathic and kind, and women who deviate from this norm, particularly in their motherhood, experience this as their failing as mothers. Gender roles can produce a lot of stress. In the female gender role, such stress is created by fear of being physically unattractive and by fear of self-assertive behaviour [24].

In the literature, many women mention that they find their premenstrual bodies ugly and unattractive [25]. Our study does not show that. We do think, however, that negative terms like ‘witch’ and ‘bitch’ that women used to describe themselves in their premenstrual period are related to the gendered stigmatisation of assertive women.

Some studies also mention positive symptoms, such as higher energy levels in the premenstrual phase, meeting with comfort and sympathy or talking with peers about the symptoms [26]. The women with PMD in our study mentioned ways of dealing with the problems, which we classified as active coping and coping by avoidance. This is also evident in current coping literature [27,28]. Many women considered PMD to be a major problem. Women with strictly somatic cognitions about causality showed a more active approach in their coping strategy, mentioned that they needed a specialist approach and searched for all possible treatments available to relieve their symptoms. They expressed a need for acknowledgement, diagnostics and somatically focussed treatment. A minority of women with integral cognitions about causality used an avoidant coping strategy. They considered their symptoms a natural part of being a woman and tried to learn how to deal with them. This self-regulation allows women to stay in control of things in their social environment [29].

### The expectations of GPs

The patients’ views and expectations contrast with the views of GPs, who consider PMD to be physiological in origin and primarily opt to give lifestyle advice and recommend women to accept symptoms [15]. Unawareness of the women’s perspectives or disregarding them may give rise to miscommunication in doctor-patient relationships. GPs need to explore the patients’ views, social consequences and related expectations. This is all the more important as some women have suicidal thoughts because of PMD. In this regard, it is very important for GPs to pursue an open communication style with their patients to explore women´s cognitions and offer personalised care. Women also expect a proper diagnosis of PMD and acknowledgement from their GPs.

We believe that PMD should be seen from the perspective of a biopsychosocial model, focussing on biomedical aspects as well as psychological and social factors that are crucial to illness and the healing process [3,30].

### Implication for daily practice

A greater awareness of PMD in general practice is needed to give women with PMD the self-esteem they are sorely missing in their contacts with GPs. A gendered perspective is necessary to deconstruct the socially constructed female identity and gender roles as a good mother, wife and always caring woman. We advocate that GPs enhance their knowledge and are competent to diagnose and treat women with PMD properly. To offer personalised care, GPs should explore women’s cognitions, experiences, coping style and the social impact of the disorder. Therefore, we strongly advise the Dutch Association of GPs to develop a guideline on PMD.

## Conclusion

This study shows that in women with PMD mental symptoms can strongly interfere with their self-perception of being good mothers and partners. These women experience having separate female identities and consider PMD a life-controlling condition. The majority use active coping strategies and a minority use avoidant coping strategies related to their cognitions about PMD. This study shows a considerable discrepancy between women’s expectations and their GPs’ views regarding PMD. Somatic, psychological and social aspects should be acknowledged. Women with PMD require understanding and acknowledgement from their GPs and their social support system.

## Supplementary Material

Supplemental Material: Example of categorisation

## Abbreviations

COREQ: Consolidated criteria for REporting Qualitative research
GP: general practitioner
ISPMD: International Society for Premenstrual Disorders
PMD: Premenstrual Disorder
PMDD: Premenstrual Dysphoric Disorder
PMS: Premenstrual Syndrome.
