# Supplementary material for: Perspectives of Dutch women on premenstrual disorder. A qualitative study exploring women’s experiences
Source: Eur J Gen Pract. 2023 Jan 30;29(1):2166033. doi: 10.1080/13814788.2023.2166033 (PMC9888467; doi:10.1080/13814788.2023.2166033)
Supplement: Supplemental Material: Example of categorisation [file IGEN_A_2166033_SM4133.docx]

**Example of categorisation**

Theme: Life-controlling condition

| ***Quote*** | ***Code*** | ***Description*** | ***Category*** |
| --- | --- | --- | --- |
| ‘If you feel … you’re really going to doubt yourself if this is indeed the case. If you’re not taken seriously, neither by your family and friends, nor by doctors, that’s quite sad.’ | Diminishing self-confidence, feeling unappreciated | Reduction of self-esteem and self-confidence, loss of interest in work | Restriction, challenges of life |
| ‘I´m an experienced person, but I suddenly started to ask myself questions.’ | Loss of concentration, overview and efficiency; making mistakes, calling in sick or losing job | Consequences for professional practice | Constraints to participation / professional satisfaction |
| ‘And so … I felt the urge to end it all.’ | Preoccupation with PMD, recurrent thoughts of not valuing life | Desperate mental state of mind | Meaning of life |
